# Centromere 17 copy number gain reflects chromosomal instability in breast cancer

**DOI:** 10.1038/s41598-019-54471-w

**Published:** 2019-11-29

**Authors:** Kyoungyul Lee, Hyun Jeong Kim, Min Hye Jang, Sejoon Lee, Soomin Ahn, So Yeon Park

**Affiliations:** 1 Department of Pathology, Seoul National University College of Medicine, Seoul, Republic of Korea; 2 Department of Pathology, Kangwon National University Hospital, Chuncheon, Kangwon Republic of Korea; 3 Department of Pathology, Seoul National University Bundang Hospital, Seongnam, Gyeonggi Republic of Korea; 4 Department of Pathology, Yeungnam University Medical Center, Daegu, Republic of Korea; 5 Precision Medicine Center, Seoul National University Bundang Hospital, Seongnam, Gyeonggi Republic of Korea

**Keywords:** Breast cancer, Tumour biomarkers, Predictive markers

## Abstract

Chromosomal instability (CIN) is known to be associated with prognosis and treatment response in breast cancer. This study was conducted to determine whether copy number gain of centromere 17 (CEP17) reflects CIN, and to evaluate the prognostic and predictive value of CIN in breast cancer. CIN status was determined by summing copy number gains of four centromeric probes (CEP1, CEP8, CEP11, and CEP16) based on fluorescence *in situ* hybridization and CIN scores were calculated using next generation sequencing data. High CIN was associated with adverse clinicopatholgical parameters of breast cancer. Among them, positive HER2 status, high Ki-67 index and CEP17 copy number gain were found to be independent predictors of high CIN. High CIN was associated with poor clinical outcome of the patients in the whole group, as well as in luminal/HER2-negative and HER2-positive subtypes. CEP17 copy number was significantly higher in the high-CIN-score group than in the low-CIN-score group. A positive linear correlation between the mean CEP17 copy number and the CIN score was found. In conclusion, CEP17 copy number was confirmed as a useful predictor for CIN in breast cancer, and high CIN was revealed as an indicator of poor prognosis in breast cancer.

## Introduction

Assessment of HER2 status using immunohistochemistry and/or *in situ* hybridization (ISH) is an essential step for selection of patients with breast cancer for HER2-targeted therapy. In dual-colored ISH of HER2, chromosome enumeration probe targeting centromere 17 (CEP17) has been employed as a control probe for correction of chromosome aneuploidy. Although the CEP17 is not a subject of interest in breast cancer, some studies have shown that a gain in the CEP17 copy number is associated with HER2 protein overexpression^1,2^. Others have reported that CEP17 copy number gain is related to the responsiveness to anthracycline-based chemotherapy^3,4^. As for its prognostic significance, it has been found to be associated with adverse clinicopathological features^5–7^ and poor prognosis in patients with breast cancer^8,9^. In a previous study, we have shown that a gain in the CEP17 copy number is an indicator of poor prognosis in patients with luminal/HER2-negative breast cancers, suggesting that CEP17 copy number gain may reflect chromosomal instability (CIN) in breast cancer^10^.

CIN is defined as a defect that frequently results in the loss or gain of a whole or part of a chromosome during cell division in malignant solid tumors^11^. Defects in chromosome cohesion, mitotic checkpoint function, centrosome copy number, kinetochore-microtubule attachment dynamics, and cell-cycle regulation are considered to be the underlying mechanisms of CIN^12^. As a hallmark of cancer, CIN contributes to tumorigenesis through the inactivation of tumor suppressor genes^13^. CIN-induced genetic changes lead to intratumoral heterogeneity, which allows tumor cells to adapt to unfavorable environments and therapeutic agents^11,14^. Tumors with high CIN are associated with poor prognoses in various cancer types, including breast cancer^15–17^. In addition to its prognostic implications on malignant tumors, CIN may be a promising predictor for treatment response^18^. Especially, high CIN has been reported to be associated with sensitivity to anthracycline^19,20^ and resistance to taxane^21,22^.

However, although CIN is known to be associated with the clinical outcome and response to chemotherapy in breast cancer patients, it is not a useful biomarker because there is no practical method for its assessment^23^. Therefore, the discovery of a correlative marker for CIN could be useful in the prognostication as well as management of breast cancer patients. In this study, we assessed the correlation between the gain in the CEP17 copy number and CIN in breast cancer to determine whether CEP17 copy number gain reflects CIN in breast cancer. The CIN status was determined with fluorescence ISH (FISH) using multiple CEP probes on the first set of breast cancer samples. In addition, we determined the prognostic and predictive value of CIN in breast cancer. Finally, we analyzed the correlation between CEP17 copy number and CIN scores, which were measured by analyzing copy number variations in next generation sequencing (NGS) data in the second subset of breast cancer patients.

## Results

### CEP copy number gain and CIN

**Figure 1:**
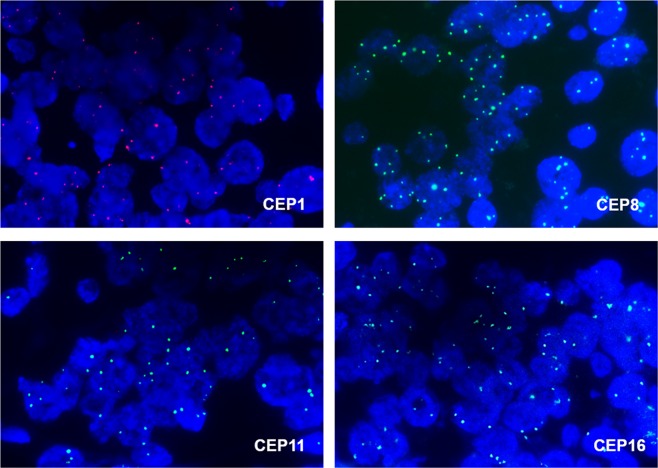
CEP copy number gain detected in fluorescence *in situ* hybridization. Representative images of CEP1, CEP8, CEP11, and CEP16 copy number gain with an increased number of three or more signals per cell.

Of the 463 cases of invasive breast cancer in the first set (Table 1), 88 (19.0%) were HER2-amplified and 375 (81.0%) were non-amplified. CEP17 status were evaluated in 460 cases and copy number gain was detected in 59 cases (12.8%). CEP17 copy number loss (mean CEP17 count <1.6) was found in three cases (0.7%). CEP1, CEP8, CEP11, and CEP16 FISH analyses were completed in 443 (95.7%), 462 (99.8%), 448 (96.8%), and 451 (97.4%) cases, respectively. According to the criteria for CEP copy number gain (mean CEP count ≥ 3), copy number gains for CEP1, CEP8, CEP11, and CEP16 were noted in 213 (48.1%), 76 (16.5%), 247 (55.1%), and 247 (54.8%) cases, respectively (Fig. 1).

**Table 1:** Baseline characteristics of the first set.

Clinicopathologic characteristics	Number of subjects (%)
**Age**	
<50 years	251 (54.2)
≥50 years	212 (45.8)
**Sex**	
Male	3 (0.6)
Female	460 (99.4)
**Histologic subtype**	
Invasive ductal carcinoma, NOS	398 (86)
Invasive lobular carcinoma	21 (4.5)
Other subtypes	44 (9.5)
**pT stage**	
pT1	198 (42.8)
pT2	238 (51.4)
pT3	19 (4.1)
pT4	8 (1.7)
**Lymph node metastasis**	
Absent	251 (54.2)
Present	212 (45.8)
Histologic grade*	
I	80 (17.9)
II	152 (34.1)
III	214 (48.0)
**Estrogen receptor**	
Positive	313 (67.6)
Negative	150 (32.4)
**Progesterone receptor**	
Positive	270 (58.3)
Negative	193 (41.7)
**Hormone receptor**	
Positive	323 (69.8)
Negative	140 (30.2)
**HER2 status**	
Negative	375 (81.0)
Positive	88 (19.0)
p53 overexpression*	
Absent	356 (77.1)
Present	106 (22.9)
**Ki-67 index**	
<20%	270 (58.3)
≥20%	193 (41.7)
**Molecular subtype**	
Luminal/HER2-negative subtype	283 (61.1)
Luminal/HER2-postive subtype	40 (8.6)
HER2-positive subtype	48 (10.4)
Triple-negative subtype	92 (19.9)
CEP17 copy number gain*	
Absent	401 (87.2)
Present	59 (12.8)

*There are some missing data.

To assess the degree of CIN, we summed the CEP copy number gains for chromosomes 1, 8, 11, and 16 in each breast cancer. One hundred thirty-two cases (28.5%) showed copy number gain for one CEP, 123 (26.6%) for two CEPs, 97 (21.0%) for three CEPs and 29 (6.3%) for all four CEPs. No gains in four CEPs were found in 82 (17.7%) cases. One hundred twenty-six (27.2%) breast cancers showing copy number gains in three or more CEPs were classified as the high-CIN group. The remaining 337 (72.8%) cases were classified as the low-CIN group.

### Association of CIN with clinicopathological parameters including CEP17 copy number gain

High CIN correlated with well-known poor prognostic parameters, including the high T stage (p = 0.007), lymph node metastasis (p = 0.010), high histological grade (p < 0.001), lymphovascular invasion (p = 0.010), negative hormone receptor status (p = 0.024), positive HER2 status (p < 0.001), p53 overexpression (p = 0.001), and high Ki-67 index (p < 0.001). As for breast cancer subtype, high CIN was more frequent in luminal/HER2-postive and HER2-positive subtypes than luminal/HER2-negative subtype (p < 0.001, p = 0.001, respectively). In addition to these accepted clinicopathological factors, the CEP17 copy number gain was clearly associated with high CIN. The proportion of CEP17 copy number gain was significantly higher in high-CIN tumors than in low-CIN tumors (27.8% vs. 7.2%; p < 0.001) (Table 2).

**Table 2:** Correlations between chromosomal instability status and clinicopathologic characteristics.

Clinicopathologic characteristics	Chromosomal instability		*p* value
	Low	High	
**Age**			0.266
<50 years	188 (55.8)	63 (50.0)	
≥50 years	149 (44.2)	63 (50.0)	
**T stage**			0.007
T1	157 (46.6)	41 (32.5)	
T2–4	180 (53.4)	85 (67.5)	
**Lymph node metastasis**			0.010
Absent	195 (57.9)	56 (44.4)	
Present	142 (42.1)	70 (55.6)	
Histologic grade*			<0.001
I & II	185 (57.3)	47 (38.2)	
III	138 (42.7)	76 (61.8)	
**Lymphovascular invasion**			0.010
Absent	203 (60.2)	59 (46.8)	
Present	134 (39.8)	67 (53.2)	
**Hormone receptor**			0.024
Positive	245 (72.7)	78 (61.9)	
Negative	92 (27.3)	48 (38.1)	
**HER2 status**			<0.001
Negative	289 (85.8)	86 (68.3)	
Positive	48 (14.2)	40 (31.7)	
p53 overexpression*			0.001
Absent	273 (81.0)	83 (66.4)	
Present	64 (19.0)	42 (33.6)	
**Ki-67 index**			<0.001
<20%	217 (64.4)	53 (42.1)	
≥20%	120 (35.6)	73 (57.9)	
**Molecular subtype**			<0.001
Luminal/HER2-negative subtype	224 (66.5)	59 (46.8)	
Luminal/HER2-postive subtype	21 (6.2)	19 (15.1)	
HER2-positive subtype	27 (8.0)	21 (16.7)	
Triple-negative subtype	65 (19.3)	27 (21.4)	
CEP17 copy number gain*			<0.001
Absent	310 (92.8)	91 (72.2)	
Present	24 (7.2)	35 (27.8)	

*There are some missing data.

In order to identify independent predictive factors for CIN, a multivariate logistic regression analysis was performed. Positive HER2 status (p = 0.021), high Ki-67 index (p = 0.027), and CEP17 copy number gain (p < 0.001) were found as independent predictors of high CIN. The odd ratios for positive HER2 status, high Ki-67 index, and CEP17 copy number gain were 1.930 (95% CI 1.105–3.372), 2.007 (95% CI 1.082–3.724), and 3.760 (95% CI 2.026–6.679), respectively (Table 3). This analysis demonstrated that CEP17 copy number gain is a strong independent predictor for high CIN.

**Table 3:** Multivariate logistic regression analysis for predictors of high chromosomal instability.

Variables	Odds ratio (95% CI)	*p* value
T stage (T1 vs. T2–4)	1.567 (0.969–2.535)	0.067
Lymph node metastasis (Absent vs. Present)	1.604 (0.965–2.668)	0.068
Histologic grade (I & II vs. III)	1.269 (0.683–2.357)	0.451
Lymphovascular invasion (Absent vs. Present)	1.190 (0.717–1.976)	0.501
Hormone receptor (Negative vs. Positive)	1.475 (0.811–2.681)	0.203
HER2 status (Negative vs. Positive)	1.930 (1.105–3.372)	0.021
p53 overexpression (Absent vs. Present)	1.548 (0.875–2.738)	0.134
Ki-67 index (<20% vs. ≥20%)	2.007 (1.082–3.724)	0.027
CEP17 copy number gain (Absent vs. Present)	3.760 (2.026–6.979)	<0.001

CI, confidence interval.

### Prognostic significance of CIN in breast cancer

**Figure 2:**
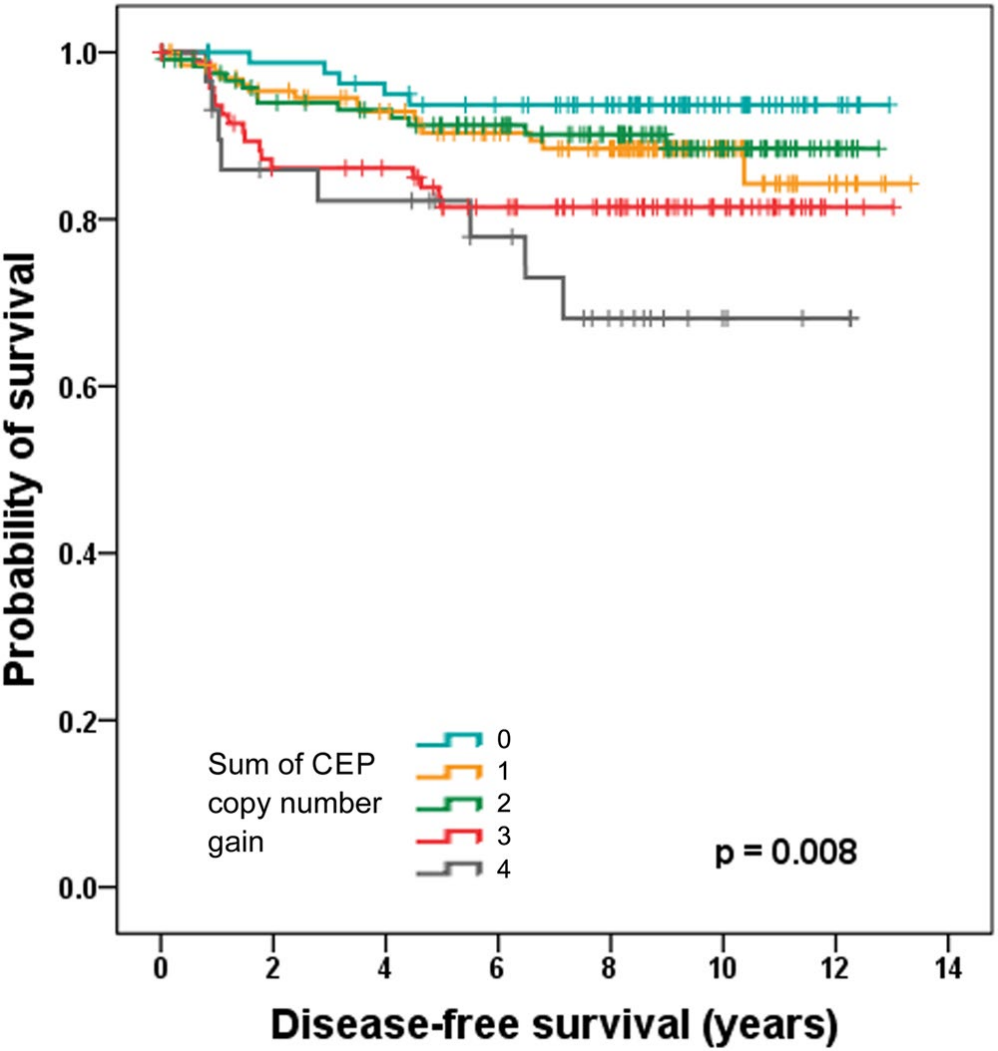
Kaplan–Meier survival analysis according to the sum of the CEP copy number gains. Disease-free survival of the patients becomes poorer as the sum of CEP copy number gains increases. Survival difference is most distinct between sum of CEP copy number gain of two and three.

**Figure 3:**
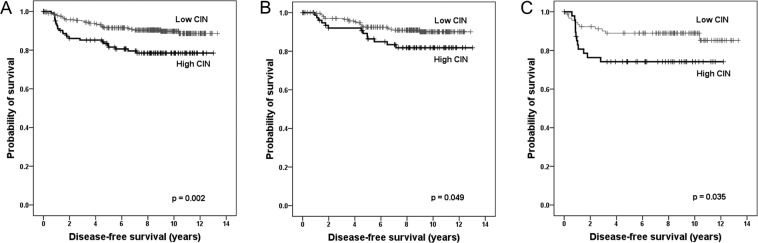
Kaplan-Meier survival analyses according to chromosomal instability status. High chromosomal instability (CIN) is a significant adverse prognostic factor in the whole group (**A**), in hormone receptor-positive tumors (**B**), and in the hormone receptor-negative tumors (**C**).

**Figure 4:**
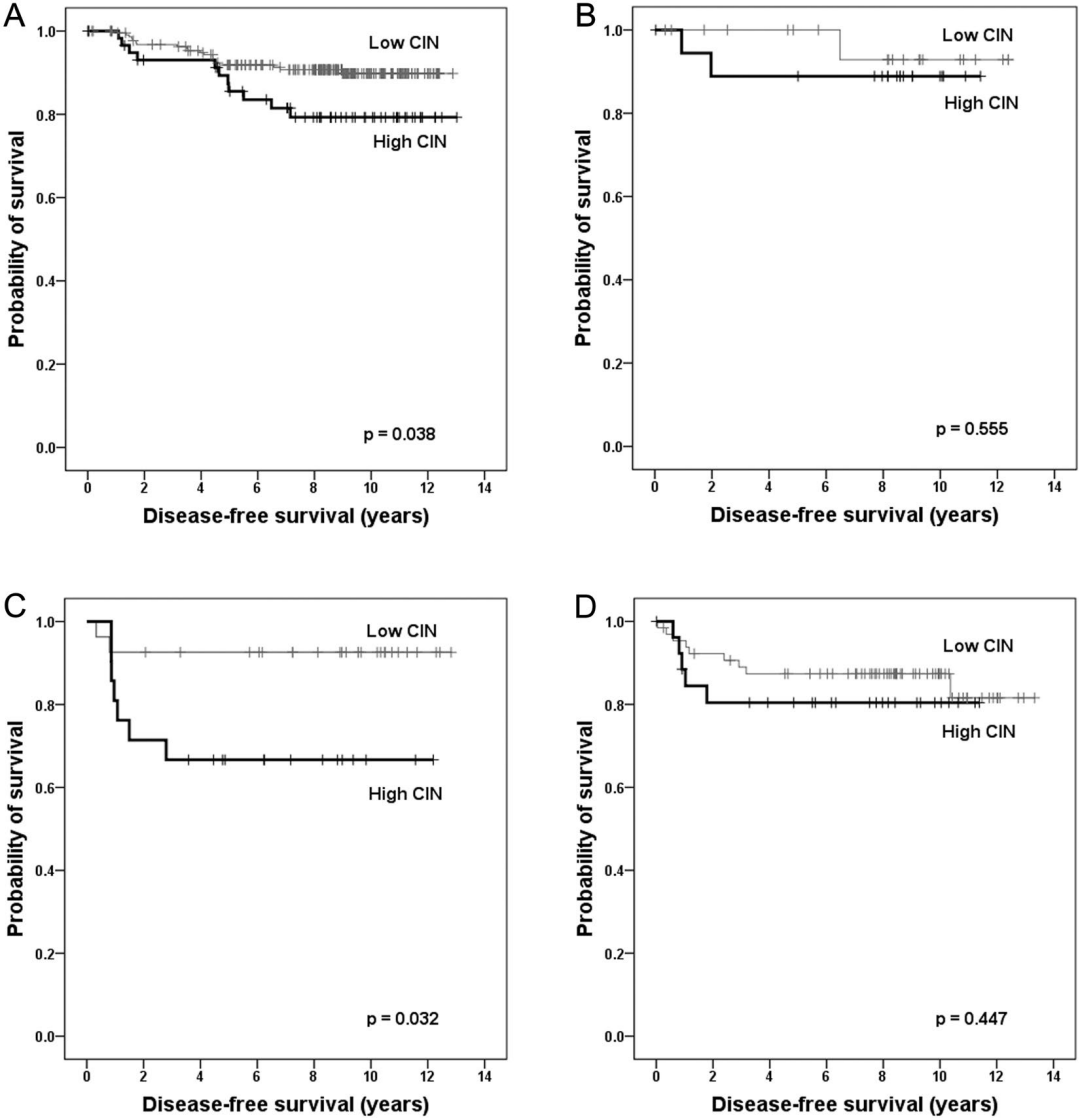
Kaplan-Meier survival analyses based on chromosomal instability status in breast cancer subtypes. Survival analyses in breast cancer subtypes shows that high chromosomal instability (CIN) is a significant indicator of poor prognosis in the luminal/HER2-negative (**A**) and HER2-positive subtypes (**C**), but it is not proven to be a prognostic factor in the luminal/HER2-positive (**B**) and triple-negative subtypes (**D**).

Next, we assessed the prognostic significance of CIN in breast cancer. According to the Kaplan–Meier survival analysis, the sum of the CEP copy number gains was significantly associated with disease-free survival and the clinical outcome of the patients deteriorates as the sum of CEP copy number gains increased (p = 0.008; Fig. 2). During the division of the samples into high-CIN and low-CIN groups, it was seen that the high-CIN group showed significantly shorter disease-free survival compared to low CIN group (p = 0.002; Fig. 3). In the subgroup based on the hormone receptor status, high CIN was associated with shortened disease-free survival time both in hormone receptor-positive and hormone receptor-negative subgroups (p = 0.049, p = 0.035, respectively; Fig. 3). Concerning breast cancer subtype, high CIN was associated with poor disease-free survival in luminal/HER2-negative and HER2-postive subtypes (p = 0.038, p = 0.032, respectively; Fig. 4). CIN status was not associated with survival of the patients in luminal/HER2-positive and triple-negative subtypes (p = 0.555, p = 0.447, respectively; Fig. 4).

Besides high CIN (p = 0.002), high T stage (p = 0.012), lymph node metastasis (p < 0.001) and lymphovascular invasion (p < 0.001) were associated with poor disease-free survival of the patients in univariate analysis (Table 4). Negative hormone receptor status tended to be associated with poor clinical outcome of the patients (p = 0.053). In the multivariate analysis, lymph node metastasis (hazard ratio, 2.528; 95% CI, 1.318–4.850; p = 0.005), lymphovascular invasion (hazard ratio, 2.037; 95% CI, 1.099–3.775; p = 0.024), negative hormone receptor status (hazard ratio, 2.002; 95% CI, 1.169–3.430; p = 0.011) and high CIN (hazard ratio, 1.813; 95% CI, 1.067–3.080; p = 0.028) were revealed as independent factors of poor prognosis (Table 4).

**Table 4:** Univariate and multivariate analyses of disease-free survival in the whole group.

Variables	Univariate analysis		Multivariate analysis	
	Hazard ratio (95% CI)	*p* value	Hazard ratio (95% CI)	*p* value
Onset age (<50 years vs. ≥50 years)	0.908 (0.538–1.533)	0.719	—	—
T stage (T1 vs. T2–4)	2.137 (1.185–3.855)	0.012	1.358 (0.740–2.494)	0.323
Lymph node metastasis (Absent vs. Present)	3.402 (1.886–6.135)	<0.001	2.528 (1.318–4.850)	0.005
Histologic grade (I & II vs. III)	1.309 (0.770–2.226)	0.320	—	—
Lymphovascular invasion (Absent vs. Present)	2.904 (1.661–5.077)	<0.001	2.037 (1.099–3.775)	0.024
Hormone receptor (Positive vs. Negative)	1.687 (0.994–2.863)	0.053	2.002 (1.169–3.430)	0.011
HER2 status (Negative vs. Positive)	1.202 (0.636–2.272)	0.571	—	—
p53 overexpression (Absent vs. Present)	1.128 (0.616–2.065)	0.696	—	—
Ki-67 index (<20% vs. ≥20%)	1.411 (0.840–2.373)	0.193	—	—
CEP17 copy number gain	1.546 (0.781–3.062)	0.211	—	—
CIN (Low vs. High)	2.270 (1.345–3.831)	0.002	1.813 (1.067–3.080)	0.028

### Association of CIN with treatment response

**Figure 5:**
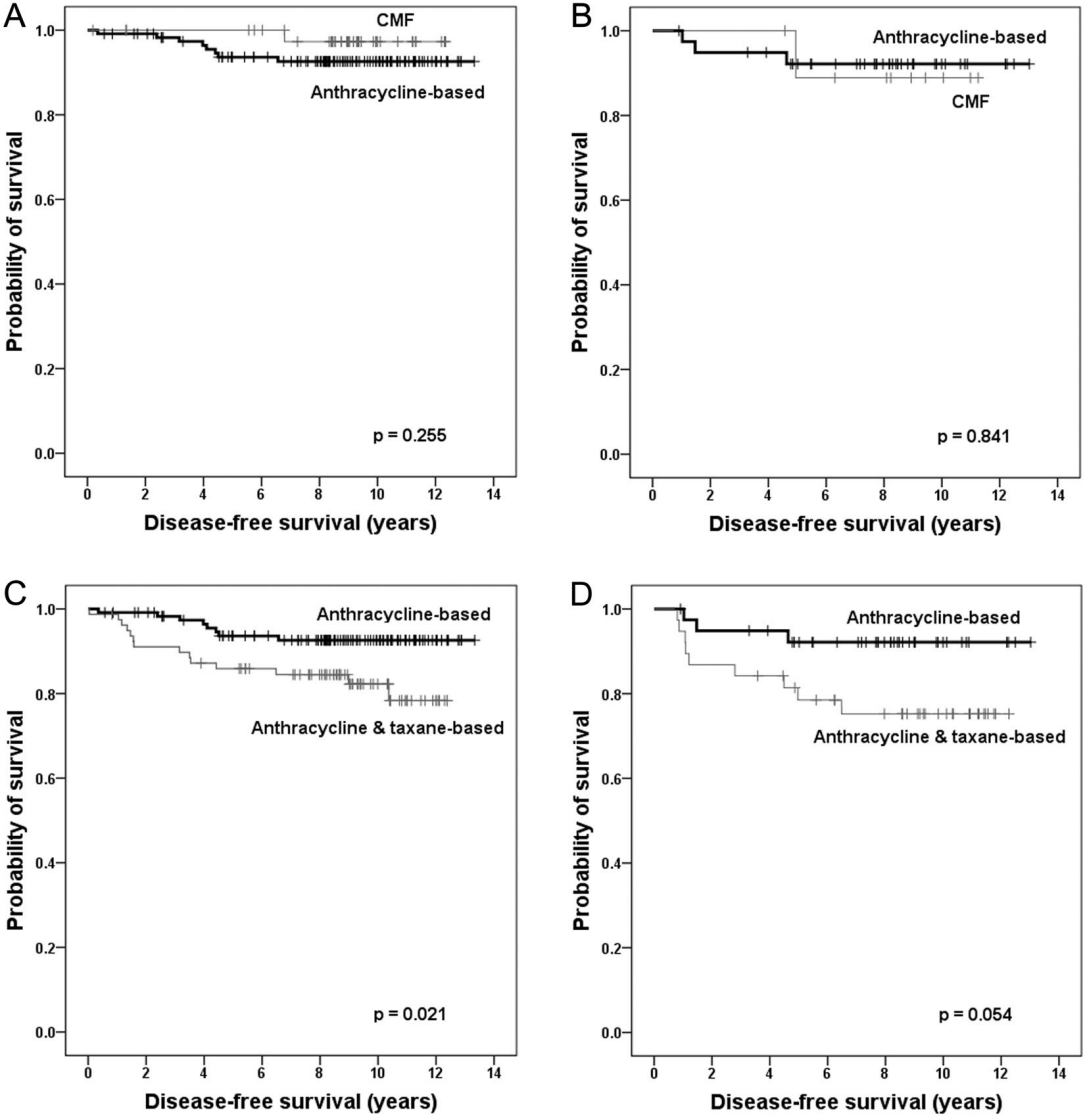
Kaplan–Meier survival analyses of disease-free survival according to the chemotherapy regimens. There are no differences in disease-free survival rates between the patients treated with anthracycline-based chemotherapy and those treated with CMF chemotherapy, in either the low- chromosomal instability (CIN) (**A**) or high-CIN group (**B**). Disease-free survival is poorer in patients treated with anthracycline & taxane-based chemotherapy than in those treated with anthracycline-based chemotherapy in low-CIN group (**C**), and tends to be poor in high CIN groups (**D**).

Of the 463 patients, 36 (7.8%) patients received neoadjuvant chemotherapy, and 329 (71.1%) received adjuvant chemotherapy. Of the 329 patients treated by adjuvant chemotherapy, 158 (48.0%) received anthracycline-based chemotherapy, 117 (35.6%) received anthracycline & taxane-based chemotherapy, and 54 (16.4%) received cyclophosphamide, methotrexate, and fluorouracil (CMF) chemotherapy. To assess the predictive value of the CIN status on anthracycline or tanxane response, difference in disease-free survival according to different chemotherapeutic regimens was investigated among the patients who received adjuvant chemotherapy. However, disease-free survival did not differ between the patients treated with anthracycline-based chemotherapy and those treated with CMF chemotherapy in either the low-CIN or high-CIN group (p = 0.255, p = 0.841, respectively; Fig. 5). Moreover, clinical outcome was worse in patients treated with anthracycline & taxane-based chemotherapy than in those treated with anthracycline-based chemotherapy in low-CIN group and tended to be poor in high-CIN groups (p = 0.021, p = 0.054, respectively; Fig. 5)

### Correlation between the CIN score and the CEP17 copy number gain

**Figure 6:**
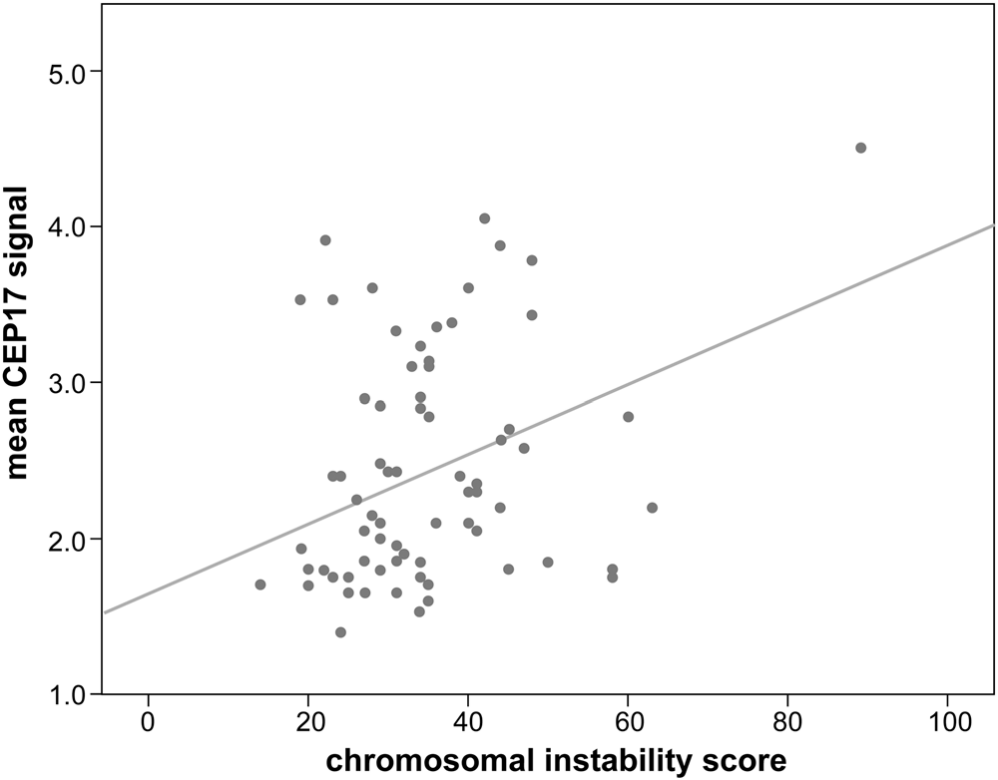
Correlation between the chromosomal instability score using next generation sequencing data and mean CEP17 copy number in the second set. A scatter dot plot shows a positive correlation (ρ = 0.353; *p* = 0.003).

The second set of 71 cases of invasive breast cancer was used for correlation of CEP17 copy number with CIN scores based on NGS. The mean CEP17 copy number ranged from 1.15 to 4.5. The CIN scores were calculated from 14 to 89 using a Z-score of the NGS data. To assess the association of CIN score with CEP17 copy number, tumors with CIN scores above the upper quartile were categorized as the high-CIN-score group and the remaining were categorized as the low-CIN-score group. The mean CEP17 copy number was higher in the high-CIN-score group than in the low-CIN-score group (2.87 ± 0.94 vs. 2.31 ± 0.65; p = 0.028). A simple regression analysis between the CIN score and the mean CEP17 signal was also used to confirm their correlation and a significant positive correlation (ρ = 0.353; p = 0.003) was found between the CIN score and the mean CEP17 copy number (Fig. 6).

## Discussion

A gain in the CEP17 copy number is a genetic change commonly observed during HER2 ISH for breast cancer and was reported in 3% to 46% of breast cancers^24^. Using a threshold of CEP17 ≥ 3.0, we detected a CEP17 copy number gain in 12.8% of breast cancers tested in this study. Although CEP17 copy number gain has been reported to be associated with poor clinical outcome^8–10^ and the responsiveness to anthracycline-based chemotherapy in patients with breast cancer^3,4^, the significance of copy number gain in CEP17, which detects non-coding peri-centromeric region of the chromosome, has not been clear. In the present study, we focused on the association of CEP17 copy number gain with CIN in breast cancer.

Using the sum of CEP copy number gains as a unique measure of CIN, we showed that high CIN correlated significantly with aggressive clinicopathological parameters, including high T stage, lymph node metastasis, high histological grade, lymphovascular invasion, negative hormone receptor status, positive HER2 status, p53 overexpression, and high Ki-67 index. The association between high CIN and aggressive clinicopathologic features of breast cancer is consistent with the results from a previous study^25^. We also showed that high CIN correlated with luminal/HER2-positive and HER2-postive subtypes. This finding can be explained by the association of distinct patterns of DNA copy number alteration with breast cancer subtypes: a “simple” type with few gains or losses in luminal A subtype, an “amplifier” type with focal high-level DNA amplifications in luminal B and HER2 subtypes, and a “complex” type characterized by numerous low-amplitude changes in triple-negative subtype^26^. However, more importantly, high CIN correlated strongly with CEP17 copy number gain. In multivariate logistic regression analysis, the CEP17 copy number gain was revealed as an independent predictor of high CIN with odd ratio of 3.760 (95% CI 2.026–6.979), which indicates an independent as well as strong association between the CEP17 copy number gain and high CIN. To overcome the limitations in assessment of CIN by FISH, we calculated the CIN scores from NGS data. We observed a higher mean CEP17 copy number in the high-CIN-score group than in the low-CIN-score group. We also identified a positive linear correlation between the mean CEP17 copy number and the CIN score. Consistent with this observation, a previous study reported an association between CEP17 copy number and CIN which was assessed using four CEPs^20^. Based on these findings, we suggest that an increase in CEP17 copy number is a practical predictor of CIN in breast cancer.

In this study, we showed that the sum of CEP copy number gains correlated strongly with the prognosis of the breast cancer patients. In an additional analysis of the dichotomized CIN status, the high-CIN group showed clearly poorer clinical outcomes than the low-CIN group. This result is consistent with previous studies showing relationship between CIN and clinical outcome of the patients with breast cancer^15–17^, although the methods for CIN measurement were different. While we determined CIN status using interphase-FISH with centromeric probes, one study employed ‘functional aneuploidy profile’ from gene expression data^15^, and two other studies used single nucleotide polymorphisms array for assessment of CIN^16,17^. In subgroup analysis, high CIN was revealed as an indicator of poor prognosis in patients with the luminal/HER2-negative subtype. In the present study, although CEP17 copy number gain was not associated with clinical outcome of the patients in this subtype (p = 0.114; data not shown), probably due to small sample size, we demonstrated in our previous study that CEP17 copy number gain is an indicator of poor prognosis only in the luminal/HER2-negative subtype of breast cancer^10^. This finding also supports that CEP17 copy number gain and CIN are closely related. Previous studies also have shown that CIN is associated with clinical outcome in luminal subtype of breast cancers^16,17^.

Our study also showed that high CIN is associated with a poor prognosis in the HER2-positive subtype of breast cancer. Smid *et al*.^17^ also showed that CIN-score was significantly associated with prognosis in HER2-postive subtype. The reason why high CIN is associated with poor prognosis in HER2-postive subtype is not clear, but in the present study, high CIN status was found to be correlated with lymph node metastasis and lymphovascular invasion in this subtype (p = 0.005, p = 0.011, respectively; data not shown). Further studies would be needed to confirm the prognostic significance of CIN and its mechanism of action on HER2-postive breast cancer.

In contrast, we observed that CIN was not a relevant prognostic factor in triple-negative subtype. Triple-negative breast cancer is characterized by complex-pattern genomes and thus, high CIN status^17,26^. High CIN generally leads to intratumoral heterogeneity, which allows tumor cells to avoid the immune system at the genetic level and leads to tumor progression^14^. However, extremely high CIN, which is found in a subset of triple-negative breast cancer, can reduce tumor viability through activation of immune surveillance. A previous study showed that extreme CIN was associated with a better prognosis in ER-negative breast cancer patients^27^. Triple-negative breast cancer is heterogeneous group of disease, and hence, the CIN would be quite variable, although on the higher side. Therefore, simple dichotomization of CIN into low or high CN groups would not provide adequate prognostic information in triple-negative breast cancer patient.

Although results have been conflicting, several studies have reported that CIN can predict the responsiveness of breast cancer patients to specific chemotherapeutic agents^18–22^. Those studies have shown that high CIN is associated with a favorable anthracycline response and taxane resistance. Since a considerable number of patients received anthracycline-based chemotherapy, anthracycline & taxane-based chemotherapy or CMF chemotherapy in this cohort, the association between anthracycline or taxane responsiveness and CIN status was analyzed. However, in comparison with CMF chemotherapy, no predictive value of high CIN in response to anthracycline-based chemotherapy was found. Similarly, the relationship between high CIN with taxane resistance was not demonstrated in this study.

There are some limitations in this study. First, although the assessment of CIN status using CEP probes is accepted as an appropriate method, the limited number of CEP probes used in this study may have affected the accuracy of the CIN measurements. However, we selected chromosomes that are known to show frequent copy number gains in breast cancer to evaluate CIN. Second, we calculated the CIN scores with targeted sequencing data confined to 170 genes, which may also influence on the accuracy of CIN measurement. Finally, as a retrospective study, the patients were treated with various chemotherapeutic agents even within same classes of anthracycline or anthracycline & taxane-based chemotherapy. To validate our findings, studies with large numbers of samples in evenly treated patients are required.

In conclusion, the degree of CIN was revealed as an independent prognostic factor for patients with breast cancer in a whole group, and high CIN was found to be a meaningful prognostic indicator in several molecular subtypes of breast cancer. In particular, this study clearly demonstrated a strong positive correlation between the CEP17 copy number and CIN in breast cancer. As CEP17 status of a tumor is readily accessible with routine HER2 ISH testing, the CEP17 copy number gain can be used as a useful predictor of high CIN. In addition to the HER2 status, CEP17 status needs to be evaluated carefully and included in HER2 ISH report.

## Methods

### Study population and samples

We used two different sets of breast cancer samples in this study. The first set consisted of a total of 463 invasive breast cancer samples (Table 1), which were consecutively resected between 2003 and 2008 at Seoul National University Bundang Hospital. These samples were used to analyze CIN using multiple CEP probes and to determine its prognostic and predictive values. The clinicopathological information was obtained from medical records and hematoxylin-and-eosin-stained sections. The following histopathological variables were recorded: T stage, N stage, histologic subtype (by WHO classification), Bloom-Richardson histological grade, and lymphovascular invasion. The second set, which was composed of 71 cases of invasive breast cancer surgically resected between 2010 and 2012, was used for correlation of CEP17 copy number with CIN scores based on NGS. A significant proportion (35.2%) of the second set consisted of mucinous carcinoma cases, which had been analyzed for another study (not published). The baseline characteristics are shown in Supplementary Table S1. The study was approved by the institutional review board of Seoul National University Bundang Hospital (Protocol # B-1609–362–106), which waived the requirement for obtaining informed consent for this study. All procedures performed in studies involving human participants were in accordance with the ethical standards of the institutional research committee and with the 1964 Helsinki declaration and its later amendments or comparable ethical standards.

### Tissue microarray construction

All the slides of each breast cancer from the first set were reviewed to select representative sections. Tissue microarrays (TMAs) of a diameter of 2 mm were constructed from representative formalin-fixed paraffin-embedded blocks (SuperBioChips Laboratories, Seoul, South Korea) for FISH.

### Immunohistochemical analysis and scoring

The expression of the estrogen receptor (ER), progesterone receptor (PR), HER2, p53 and Ki-67 was evaluated in the representative tumor sections of the surgical specimens at the time of diagnosis. With regards to the cases with missing data, immunohistochemical staining on representative tissue sections was carried out in a BenchMark XT autostainer (Ventana Medical Systems, Tucson, AZ) using an UltraView detection kit (Ventana Medical Systems). The following antibodies were used: anti-ER (1:100; clone SP1; LabVision, Fremont, CA), anti-PR (1:70; PgR 636; Dako, Carpinteria, CA), anti-HER2 (ready to use; 4B5; Ventana Medical Systems), anti-p53 (1:600; D07; Dako), and anti-Ki-67 (1:250; MIB-1; Dako).

A tumor was regarded as positive for ER or PR if it showed at least 1% positive nuclear staining with the relevant antibody. A tumor was considered as HER2 positive, if it was 3 + on immunohistochemistry or if gene amplification was seen on FISH. Nuclear staining in 10% or more of the tumor cells was considered positive for p53. Nuclear staining in 20% or more of the tumor cells was considered an indication of high Ki-67 proliferation index.

Immunohistochemical expression of the standard biomarkers were used to categorize the tumor samples into breast cancer subtypes. Breast cancer subtypes were categorized according to the criteria used in our previous study^10^: luminal/HER2-negative subtype (ER+ and/or PR+, HER2-), luminal/HER2-positive subtype (ER+ and/or PR+, HER2+), HER2-positive subtype (ER-, PR-, HER2+), and triple-negative subtype (ER-, PR-, HER2-).

### Fluorescence *in situ* hybridization

To identify the HER2 status and CEP17 copy number in each case, HER2 FISH (PathVysion assay, Abbott Molecular, Downers Grove, IL) was performed on TMAs of the first set and all tissue sections of the second set. FISH using CEP1 [Vysis CEP1 (D1Z5) SpectrumOrange Probe, Abbott Molecular], CEP8 [Vysis CEP8 (D8Z2) SpectrumGreen Probe, Abbott Molecular], CEP11 [Vysis CEP11 (D11Z1) SpectrumGreen Probe, Abbott Molecular], and CEP16 probe [Vysis CEP16 (D16Z3) SpectrumGreen Probe, Abbott Molecular] was performed on TMAs to assess CIN. These CEP probes around the centromere have been reported to show frequent copy number gains in breast cancer^17,23,28^.

Briefly, 4 μm deparaffinized tissue sections were incubated in pretreatment solution (Abbott Molecular) at 80 °C for 30 min and then, in protease solution (Abbott Molecular) at 37 °C for 20 min. Probes were diluted in tDen-Hyb-2 hybridization buffer (Insitus Biotechnologies, Albuquerque, NM). The probes and the DNA from the tissue sections were denatured together by incubating them for 5 min at 73 °C in HYBrite™ (Abbott Molecular), and then hybridized for 16 h at 37 °C. Post-hybridization washes were performed according to the manufacturer’s protocol. The mounted slides were viewed using a fluorescence microscope.

### Definition of HER2 status, CEP copy number gain and CIN

HER2 status was evaluated according to 2013 ASCO/CAP guidelines. HER2 copy number of 6 or higher per cell or a HER2:CEP17 ratio of 2 or higher was considered as amplified. HER2/CEP17 ratios <2 and HER2 copy numbers between 4 to 6 signals per cell were classified as equivocal. HER2 copy numbers <4 signals per cell and HER2/CEP17 ratios <2 were considered as non-amplified^29^. In this study, HER2-equivocal cases were regarded as HER2-negative for statistical analyses.

The number of signals for each CEP probe was counted in at least 20 non-overlapping tumor nuclei. The mean CEP counts per cell for chromosomes 1, 8, 11, 16, and 17 were calculated. CEP copy number gain was defined as a mean CEP count of ≥3.0, as defined for CEP17 in our earlier study^10^. A mean CEP count of <1.6 was defined as CEP copy number loss.

Aneuploidy is a consequence of CIN, and performing FISH using multiple CEP probes is accepted as an appropriate method to assess the degree of CIN^30^. As CEP copy number loss was rarely found only in CEP8 (13 cases, 2.8%), CIN status was determined by summing the copy number gains for CEP1, CEP8, CEP11, and CEP16 in each case. A high-CIN tumor was defined as a tumor with copy number gains in at least three CEPs. Copy number gain in one or two CEPs or no copy number gain were regarded as low-CIN.

### Determination of CIN score with NGS

Genomic DNA was extracted from formalin-fixed paraffin-embedded tissue samples. DNA library preparation and target enrichment were performed with the SureSelectXT Target Enrichment Kit (Agilent Technologies, Santa Clara, CA). Deep targeted sequencing was performed with a cancer gene panel that included 170 cancer driver genes (Supplementary Table S2). Target region bases were sequenced for each sample using the HiSeq. 2500 system (Illumina, San Diego, CA), achieving average coverage depth 715 × (Macrogen Inc., Seoul, Republic of Korea).

The adapter sequences were eliminated with cutadapt^31^. The reads were aligned to the reference genome (GRCh37/hg19) using Burrows–Wheeler Aligner MEM (BWA-MEM)^32^. Poorly mapped reads (mapping quality below 20) and duplicated reads were removed with SAMtools version 1.3.1 and MarkDuplicates (version 2.2.4), respectively. The base quality of the deduplicated reads was recalibrated with GATK BaseRecalibrator. To estimate the degree of CIN, we calculated the Z-score of the normalized number of reads in 2,897 predefined regions in each sample and scored them by counting the number of regions with |Z| > 3.

### Statistical analysis

All statistical analyses were performed with the statistical package SPSS version 15.0 (SPSS Inc., Chicago, IL). Pearson’s χ^2^ test was used to compare categorical variables between groups. A simple regression analysis was used to detect linear correlations between variables. The Mann–Whitney U test was used to compare continuous variables between two groups. A multivariate logistic regression analysis was used to detect independent predictive factors for CIN. The odds ratios and 95% confidence intervals (CIs) were calculated for the significant variables. For the survival analyses, Kaplan–Meier survival curves were generated and compared with the log rank test. A Cox proportional hazards regression model was used for the multivariate analysis with a backward stepwise selection method. Hazard ratios and 95% CIs were calculated for the significant variables. p values < 0.05 were considered statistically significant, and all reported p values are two-sided.

## Supplementary information

Supplementary Table 1 and 2

## Data Availability

The datasets used and/or analyzed during the current study are available from the corresponding author on reasonable request.
