# Supplementary material for: Centromere 17 copy number gain reflects chromosomal instability in breast cancer
Source: Sci Rep. 2019 Nov 29;9:17968. doi: 10.1038/s41598-019-54471-w (PMC6884473; doi:10.1038/s41598-019-54471-w)
Supplement: Supplementary file 1 — Supplementary Table 1 and 2 [file 41598_2019_54471_MOESM1_ESM.docx]

***SUPPLEMENTARY TABLES***

**Centromere 17 copy number gain reflects chromosomal instability in breast cancer**

Kyoungyul Lee^1,2^, Hyun Jeong Kim^3^, Min Hye Jang^4^, Sejoon Lee^5^, Soomin Ahn^3^, So Yeon Park^1,3^

^1^Department of Pathology, Seoul National University College of Medicine, Seoul, Republic of Korea;^2^Department of Pathology, Kangwon National University Hospital, Chuncheon, Kangwon, Republic of Korea; ^3^Department of Pathology, Seoul National University Bundang Hospital, Seongnam, Gyeonggi, Republic of Korea; ^4^Department of Pathology, Yeungnam University Medical Center, Daegu, Republic of Korea; ^5^Precision Medicine Center, Seoul National University Bundang Hospital, Seongnam, Gyeonggi, Republic of Korea

**Supplementary Table S1. Baseline characteristics of the second set**

| **Clinicopathologic characteristics** | **Number of subjects (%)** |
| --- | --- |
| **Age** |  |
| < 50 years | 37 (52.1) |
| ≥ 50 years | 34 (47.9) |
| **Sex** |  |
| Male | 0 (0) |
| Female | 71 (100) |
| **Histologic subtype** |  |
| Invasive ductal carcinoma, NOS | 44 (62) |
| Mucinous carcinoma | 25 (35.2) |
| Metaplastic carcinoma | 2 (2.8) |
| **pT stage** |  |
| pT1 | 35 (49.3) |
| pT2 | 28 (39.4) |
| pT3 | 8 (11.3) |
| **Lymph node metastasis** |  |
| Absent | 48 (67.6) |
| Present | 23 (32.4) |
| **Histologic grade** |  |
| I | 17 (23.9) |
| II | 28 (39.4) |
| III | 26 (36.6) |
| **Estrogen receptor** |  |
| Positive | 60 (84.5) |
| Negative | 11 (15.5) |
| **Progesterone receptor** |  |
| Positive | 48 (67.6) |
| Negative | 23 (32.4) |
| **Hormone receptor** |  |
| Positive | 60 (84.5) |
| Negative | 11 (15.5) |
| **HER2 status** |  |
| Negative | 61 (85.9) |
| Positive | 10 (14.1) |
| **p53 overexpression** |  |
| Absent | 57 (80.3) |
| Present | 14 (19.7) |
| **Ki-67 index** |  |
| <20% | 42 (59.2) |
| ≥20% | 29 (40.8) |
| **Breast cancer subtype** |  |
| Luminal/HER2-negative subtype | 56 (78.9) |
| Luminal/HER2-postive subtype | 4 (5.6) |
| HER2-positive subtype | 5 (7) |
| Triple-negative subtype | 6 (8.5) |
| **CEP17 copy number gain** |  |
| Absent | 54 (76.1) |
| Present | 17 (23.9) |

**Supplementary Table S2. List of 170 cancer-related genes in targeted sequencing**

| ABL1 | BCL2 | CDKN1B | ERBB3 | FLCN | JAK3 | MEN1 | NOTCH3 | PPARG | SMAD4 |
| --- | --- | --- | --- | --- | --- | --- | --- | --- | --- |
| ABL2 | BRAF | CDKN2A | ERBB4 | FLT1 | KDR | MET | NOTCH4 | PTCH1 | SMARCA4 |
| AKT1 | BRCA1 | CDKN2B | ERCC2 | FLT3 | KIT | MITF | NPM1 | PTEN | SMARCB1 |
| AKT2 | BRCA2 | CDKN2C | ERG | FLT4 | KMT2A | MLH1 | NRAS | RAB35 | SMO |
| AKT3 | BRD2 | CEBPA | ERRFI1 | FOXL2 | KRAS | MPL | NTRK1 | RAD50 | SRC |
| ALK | BRD3 | CHEK2 | ESR1 | GNA11 | MAP2K1 | MSH2 | NTRK2 | RAF1 | STK11 |
| APC | BRD4 | CREBBP | ETV1 | GNAQ | MAP2K2 | MSH6 | NTRK3 | RARA | SYK |
| AR | CBFB | CRKL | ETV4 | GNAS | MAP2K4 | MTOR | NUTM1 | RB1 | TET2 |
| ARAF | CCND1 | CSF1R | ETV5 | HDAC9 | MAP3K1 | MYC | PDGFB | RET | TMPRSS2 |
| ASXL1 | CCND2 | CTNNB1 | ETV6 | HGF | MAP3K4 | MYCN | PDGFRA | RHEB | TOP2A |
| ATM | CCND3 | DDR1 | EWSR1 | HRAS | MAPK1 | MYD88 | PDGFRB | RICTOR | TP53 |
| ATR | CCNE1 | DDR2 | EZH2 | IDH1 | MAPK3 | NF1 | PIK3CA | RNF43 | TSC1 |
| AURKA | CDH1 | DNMT3A | FBXW7 | IDH2 | MAPK8 | NF2 | PIK3CB | ROS1 | TSC2 |
| AURKB | CDK12 | DOT1L | FGFR1 | IGF1R | MCL1 | NFKBIA | PIK3CD | RSPO1 | VHL |
| AURKC | CDK4 | EGFR | FGFR2 | IGF2 | MDM2 | NKX2-1 | PIK3R1 | RSPO2 | WT1 |
| AXL | CDK6 | EPHA3 | FGFR3 | JAK1 | MDM4 | NOTCH1 | PIK3R2 | RUNX1 | XPO1 |
| BAP1 | CDKN1A | ERBB2 | FGFR4 | JAK2 | MED12 | NOTCH2 | POLE | SMAD2 | ZNRF3 |
